# Draft Genome Sequence of *Enterococcus faecalis* ATCC BAA-2128

**DOI:** 10.1128/genomeA.00575-17

**Published:** 2017-07-06

**Authors:** Catherine Putonti, Laurynas Kalesinskas, Evan Cudone, Kathleen C. Engelbrecht, David W. Koenig, Alan J. Wolfe

**Affiliations:** a Bioinformatics Program, Loyola University Chicago, Chicago, Illinois, USA; b Department of Biology, Loyola University Chicago, Chicago, Illinois, USA; c Department of Computer Science, Loyola University Chicago, Chicago, Illinois, USA; d Department of Microbiology and Immunology, Stritch School of Medicine, Health Sciences Division, Loyola University Chicago, Maywood, Illinois, USA; e Corporate Research and Engineering, Kimberly-Clark Corporation, Neenah, Wisconsin, USA

## Abstract

While a part of the native gut microflora, the Gram-positive bacterium *Enterococcus faecalis* can lead to serious infections elsewhere in the body. The draft genome of *E. faecalis* strain ATCC BAA-2128, isolated from piglet feces, was examined. This draft genome consists of 42 contigs, 12 of which exhibit homology to annotated plasmids.

## GENOME ANNOUNCEMENT

As part of an attempt to generate complete genomes for strains in the ATCC collection, we report the genome sequence and annotation of *Enterococcus faecalis* ATCC BAA-2128, isolated from piglet feces. Isolated as part of a study assessing the performance benefits of diets supplemented with copper, zinc, or antibiotics, the *E. faecalis* ATCC BAA-2128 strain is resistant to copper, erythromycin, and tetracycline (1).

The strain was purchased from ATCC and grown on 5% sheep blood agar (BD BBL prepared plated media) under 5% CO_2_ at 35°C for 48 h. To extract genomic DNA, the cells were resuspended in 0.5 mL of DNA extraction buffer (20 mM Tris-Cl, 2 mM EDTA, 1.2% Triton X-100, pH 8), followed by the addition of 50 µL of lysozyme (20 mg/mL), 30 µL of mutanolysin, and 5 µL of RNase (10 mg/mL). After incubation at 37°C for 1 h, 80 μL of 10% SDS and 20 µL of proteinase K were added and incubated for 2 h at 55°C. Then, 210 µL of 6M NaCl and 700 μL of phenol-chloroform were added. After a 30-min incubation with rotation, the solutions were centrifuged at 13,500 rpm for 10 min, and the aqueous phase was extracted. An equivalent volume of isopropanol was added, and the solution was centrifuged at 13,500 rpm for 10 min after a 10-min incubation. The supernatant was decanted, and the DNA pellet was precipitated using 600 μL of 70% ethanol. Following ethanol evaporation, the DNA pellet was resuspended in Tris-EDTA (TE) and stored at −20°C.

Genomic DNA was diluted in water to a concentration of 0.2 ng/µL, as measured by a fluorometric-based method (Life Technologies, Inc.), and 5 μL were used to obtain a total of 1 ng of input DNA for library preparation (Nextera XT DNA Library preparation kit). The library was sequenced on the MiSeq sequencer (Illumina) using the MiSeq version 2 reagent kit (500 cycles), producing 1,751,626 paired-end reads. Reads were trimmed, removing adapter sequences and phiX contaminants, using BBDuk from the BBMap package (http://sourceforge.net/projects/bbmap). Trimmed reads were assembled using SPAdes version 3.5 (2), followed by scaffolding with SSPACE (3). The draft genome consists of 42 contigs (*N*_50 _= 232,593 bp) with an average coverage of 265.7×. The genome size was 2,964,736 bp with an observed G+C content of 37.41%. BLASTn queries to the NR/NT database revealed that 12 of these contigs show greatest homology to plasmid sequences of other *E. faecalis* strains and enterococci, including three putative complete plasmids that are 21, 31, and 35 kbp in length. Annotations were produced using the software tool Peasant (4). Six rRNAs, 52 tRNAs, and 2,852 protein-coding sequences were detected. Two confirmed and two putative clustered regularly interspaced short palindromic repeat sequences (CRISPRs) were found (5). CRISPR spacer sequences were then queried via BLASTn to viral sequences within the NR/NR database. While not all spacer sequences produced hits, BLASTn did identify spacer sequence homology to the genomes of four phages: VPE25, vB_EfaS_IME196, SAP5, and IME-EF4.

### Accession number(s).

The draft whole-genome project for *E. faecalis* ATCC BAA-2128 has been deposited at DDBJ/EMBL/GenBank under accession number NAQY00000000. Raw sequence reads were deposited at DDBJ/EMBL/GenBank under accession number SRR5363781.
